# Modification by SUMOylation Controls Both the Transcriptional Activity and the Stability of Delta-Lactoferrin

**DOI:** 10.1371/journal.pone.0129965

**Published:** 2015-06-19

**Authors:** Adelma Escobar-Ramirez, Anne-Sophie Vercoutter-Edouart, Marlène Mortuaire, Isabelle Huvent, Stephan Hardivillé, Esthelle Hoedt, Tony Lefebvre, Annick Pierce

**Affiliations:** Unité de Glycobiologie Structurale et Fonctionnelle, UMR 8576 CNRS, Université des Sciences et Technologies de Lille, FR3688 CNRS FRABio, Villeneuve d'Ascq, France; Virginia Commonwealth University, UNITED STATES

## Abstract

Delta-lactoferrin is a transcription factor, the expression of which is downregulated or silenced in case of breast cancer. It possesses antitumoral activities and when it is re-introduced in mammary epithelial cancer cell lines, provokes antiproliferative effects. It is posttranslationally modified and our earlier investigations showed that the *O*-GlcNAcylation/phosphorylation interplay plays a major role in the regulation of both its stability and transcriptional activity. Here, we report the covalent modification of delta-lactoferrin with the small ubiquitin-like modifier SUMO-1. Mutational and reporter gene analyses identified five different lysine residues at K13, K308, K361, K379 and K391 as SUMO acceptor sites. The SUMOylation deficient M5S mutant displayed enhanced transactivation capacity on a delta-lactoferrin responsive promoter, suggesting that SUMO-1 negatively regulates the transactivation function of delta-lactoferrin. K13, K308 and K379 are the main SUMO sites and among them, K308, which is located in a SUMOylation consensus motif of the NDSM-like type, is a key SUMO site involved in repression of delta-lactoferrin transcriptional activity. K13 and K379 are both targeted by other posttranslational modifications. We demonstrated that K13 is the main acetylation site and that favoring acetylation at K13 reduced SUMOylation and increased delta-lactoferrin transcriptional activity. K379, which is either ubiquitinated or SUMOylated, is a pivotal site for the control of delta-lactoferrin stability. We showed that SUMOylation competes with ubiquitination and protects delta-lactoferrin from degradation by positively regulating its stability. Collectively, our results indicate that multi-SUMOylation occurs on delta-lactoferrin to repress its transcriptional activity. Reciprocal occupancy of K13 by either SUMO-1 or an acetyl group may contribute to the establishment of finely regulated mechanisms to control delta-lactoferrin transcriptional activity. Moreover, competition between SUMOylation and ubiquitination at K379 coordinately regulates the stability of delta-lactoferrin toward proteolysis. Therefore SUMOylation of delta-lactoferrin is a novel mechanism controlling both its activity and stability.

## Introduction

Lactoferrins exist as different variants due to gene polymorphisms, post-transcriptional and post-translational modifications [1]. The two main isoforms are secreted lactoferrin (Lf) and its nucleocytoplasmic counterpart, delta-lactoferrin (ΔLf) [2,3,4]. Their expression is downregulated or silenced in cancer cells [3,5]. In breast cancers, significantly lower levels of Lf and/or ΔLf correlated with more advanced disease and an unfavorable prognosis [5,6]. This downregulation is mainly due to genetic and epigenetic modifications that have been found on the *Lf* gene in some forms of cancer [7,8]. ΔLf mRNAs derive from the transcription of the *Lf* gene at the alternative P2 promoter leading, after translation, to a 73 kDa intracellular protein [4]. Although its subcellular distribution is mainly cytoplasmic, confocal microscopy analyses have clearly shown that ΔLf targets the nucleus [4]. Thus, we showed that ΔLf possesses a functional bipartite NLS motif in the C-terminal lobe [9]. ΔLf is capable of binding DNA but the location of its DNA binding domain is not known. Two regions of Lf in which a strong concentration of positive charges were found could be good candidates [10]. ΔLf exhibits antitumoral activities and we previously showed that overexpression of ΔLf leads to cell cycle arrest at the G1/S transition and apoptosis [11,12]. ΔLf mainly exerts its anti-proliferative and pro-apoptotic activities *via* its role as a transcription factor. Indeed, ΔLf transactivates different target genes such as *Skp1*, *DcpS*, *Bax*, *SelH*, *GTF2F2* and *UBE2E1* [9,12–14]. A genome-wide pathway analysis and our quantitative proteomic analysis showed that the re-introduction of Lf isoforms in cancerous cells modified essential genes and/or signaling networks responsible mainly for cell survival, apoptosis and RNA processing [14,15].

Since ΔLf has a variety of target genes and is involved in the control of cell homeostasis, modifications in its activity or concentration may have profound consequences. Its transcriptional activity is controlled by posttranslational modifications (PTM) among which *O*-GlcNAcylation is a key link between nutrient sensing and signaling. It notably regulates gene activation due to *O*-GlcNAc cycling on gene-specific transcription factors and components of the basal transcriptional machinery (reviewed in [16]). The targeting of serine S10 by *O*-GlcNAcylation negatively regulates ΔLf transcriptional activity whereas phosphorylation increases it [17]. Deglycosylation leads to DNA binding and a basal transactivation level which was markedly enhanced when phosphorylation was present at S10. ΔLf possesses a functional PEST sequence which drives the protein to its proteasomal degradation after polyubiquitination of the K379 and/or K391 lysine residues. ΔLf stability is also under the control of *O*-GlcNAcylation. Indeed, *O*-GlcNAcylation at S10 protects ΔLf from polyubiquitination increasing its half-life, whereas phosphorylation favours its proteasomal degradation [17].

Recently, we discovered that ΔLf is also modified by SUMOylation. The small ubiquitin-related modifier (SUMO) is involved in many aspects of cell function and affects pathways as diverse as DNA repair, cell cycle, transcriptional regulation, RNA processing, and cell signaling [18,19]. At a molecular level, SUMOylation of target proteins alters their protein-protein interactions, localization, stability or/and activity [20]. Many transcription factors are targeted by SUMOylation and in most cases SUMOylation triggers transcriptional repression by recruiting transcriptional co-repressors, such as histone deacetylases [21–23]. Four different SUMO isoforms (SUMO-1-4) have been identified in higher eukaryotes, although only SUMO-1-3 seem to be covalently attached to proteins. SUMO-2 and SUMO-3 share 96% identity, and both have approximately 46% identity with SUMO-1. The attachment of SUMO is a multi-step process analogous to that of ubiquitin. Thus, the SUMO pathway is mediated by SUMO-activating enzymes (E1), a unique SUMO-conjugating enzyme (E2) called Ubc9 and SUMO-ligases (E3). SUMOylation is a highly dynamic process which can be reversed by the activity of SUMO-specific isopeptidases (SENPs) [24].

SUMOs are conjugated to lysine residues in a ΨKXE/D sequence where Ψ is a large hydrophobic amino acid residue and X represents any amino acid [25]. This motif is sufficient by itself to mediate a direct interaction with Ubc9 [25,26]. Extended SUMO consensus motifs such as the negatively charged amino acid-dependent SUMO motif (NDSM) constituted by an acidic patch downstream of the ΨKXE/D motif, the phosphorylation-dependent SUMO motif (PDSM) that includes a phosphorylation site downstream of the consensus core motif and the hydrophobic cluster SUMOylation motif (HCSM) that contains several hydrophobic residues located N-terminal to the core motif have been described to promote substrate SUMOylation *via* additional interaction with Ubc9 [27–29]. Moreover, several proteins are also modified at other sites and until now it is not known how these non-consensus sites are recognized. However, substrates with a SUMO-interacting motif (SIM) could be SUMOylated within a non-consensus SUMO motif [30] and, as shown for the Death domain-associated protein 6 Daxx, phosphorylation of SIMs enhances SUMO-1 binding and conjugation [31]. SUMO-1 can be attached either to a single or to multiple lysine residues within a target protein leading either to mono- or multi-SUMOylation respectively, whereas chain formation is attributed to SUMO-2/3 [32]. However, [27] identified the human Topoisomerase I as a poly-SUMO-1 target. On the other hand, SUMO-1 may be attached to lysine residues within SUMO-2/3 chains, thereby preventing their elongation and acting therefore as a SUMO chain terminator [32,33]. Recently, mixed SUMO/ubiquitin chains have been reported [34].

Crosstalk between the SUMOylation, ubiquitination, and acetylation pathways is crucial for the regulation of protein activity and/or stability since these modifications may have different, sometimes opposing consequences [35]. Thus, SUMOylation can stabilize proteins by competing with ubiquitin [36,37]. However, heterogeneous SUMO2/3-ubiquitin chains were found on IκBα and PLM (promyelocytic leukemia) protein, contributing to their optimal proteosomal degradation [38]. Switches between SUMOylation and acetylation have also been reported for several proteins. SUMOylation of the myocyte-specific enhancer factor 2A (MEF2A) inhibits its transcriptional activity whereas acetylation increases it [39]. A similar SUMO to acetyl switch has also been described for the hypermethylated in cancer 1 protein (HIC1) [40].

Here we demonstrate that the stability and transcriptional activity of ΔLf are regulated by SUMOylation, which provides a novel regulatory mechanism for controlling ΔLf function. We identified the major SUMO and acetylation acceptor sites and we evaluated the impact of the SUMOylation/ubiquitin and the SUMOylation/acetylation interplays.

## Experimental Section

### Cell culture, transfection, and reagents

HEK-293 cells (ATC CRL-1573) were grown in monolayers and transfected (1 μg of DNA for 1 x 10^6^ cells) using DreamFect (OZ Biosciences, Marseille, France) as described [17].The amounts of ΔLf expression vectors were adjusted to maintain ΔLf amounts similar to those found in normal breast epithelial NBEC cells [5,6]. Transfections were done in triplicate (n ≥ 5). Cell viability was assessed by counting using Trypan blue 0.4% (v/v). To measure the ΔLf turnover rate indirectly, we performed incubations with cycloheximide, a potent inhibitor of *de novo* protein synthesis [41,42]. Cells were transfected with either ΔLf (WT), the SUMO mutant constructs or null vector (NV) then incubated with fresh medium supplemented by 10 μg/mL cycloheximide (CHX) for 0–150 min 24 h post transfection as described [17]. Inhibition of proteasome was performed by incubating cells with a 10 μM concentration of the proteasomal inhibitor MG132 for 2 h prior to lysis as described [17]. Inhibition of histone deacetylases was performed by incubating cells with Trichostatin A (TSA) at 15 ng/mL (TSA treated cells) or not overnight. Cell culture reagents were from Lonza. Other reagents were from Sigma.

### Plasmid preparation

pGL3-S1^Skp1^-Luc [9] and p3xFLAG-CMV10-ΔLf (WT) [17] were constructed as described. p3xFLAG-CMV10 (Sigma, St Louis, MO, USA) was used as a null vector (NV). The hemagglutinin A-Ubiquitin (HA-Ub) expression vector was a gift from Dr. C. Couturier (UMR-CNRS 8161, IBL, Lille, France). The psG5-His-SUMO-1 (His-SUMO-1), the pcDNA3.1-His-SUMO-2/3 (SUMO2/3) and the pcDNA3-SENP2-SV5 (SENP2) expression vectors were kind gifts from Dr. D. Leprince (UMR-CNRS 8161, IBL, Lille, France). All plasmids were purified using the QIAprep Spin Miniprep Kit (Qiagen Germantown, MD) according to the manufacturer’s specifications.

### Site-directed mutagenesis

Mutants were generated using the QuikChange Site-directed Mutagenesis Kit (Stratagene, Garden Grove, CA) according to the manufacturer’s instructions with p3xFLAG-CMV10-ΔLf as template and primer pairs listed in S1 Table. The constructs in which several sites were mutated were done sequentially. Following sequence verification, positive clones were used directly in transfection.

### Ubc9 knockdown

HEK-293 cells (2 x 10^6^ cells in 100-mm dish) were transfected with RNAiMax (Life Technologies), according to the manufacturer’s instructions, using 5 nM of siRNAs targeting Ubc9 (Hs_UBE2I_8 FlexiTube siRNA, Qiagen) or a scrambled control sequence (siCtrl) (Qiagen). Cells were harvested 48 h post-transfection and lysed. Cell extracts were assayed for Ubc9 content and SUMOylation levels.

### Reporter gene assays

Reporter gene assays were performed using the pGL3-S1^Skp1^-Luc reporter vector containing a single ΔLfRE and the ΔLf-expression vector (WT), different ΔLf SUMO mutant constructs or a null vector (NV). HEK-293 cells were synchronized overnight in medium containing 1% FCS before being transfected (250 ng of DNA for 2 x 10^5^ cells: 50 ng of reporter vector and 200 ng of ΔLf, SUMO mutants or null vector) using DreamFect (OZ Biosciences, Marseille, France) as described in [17]. Reporter gene assays were also performed in the presence of psG5-His-SUMO-1 (200 ng of DNA) or either in the presence of pcDNA3-SENP2-SV5 expression vectors (200 ng of DNA) or TSA (15 ng/mL, overnight) with their respective controls. Reporter gene assays on Ubc9 knockdown cells were performed in two steps. Cells were siUbc9/siCtrl (5 nM) transfected in serum-free medium which was supplemented 4 h post transfection with 1% FCS. Twenty hours later cells were transfected with WT or mutant constructs and the reporter gene. Cell lysates were assayed using a luciferase assay kit (Promega) in a Tristar multimode microplate reader LB 941 (Berthold Technologies, Bad Wildbab, Germany). Basal luciferase expression was assayed using a null vector and was determined for each condition. Relative luciferase activities were normalized to basal luciferase expression and ΔLf content as in [12] and expressed as a percentage; 100% corresponds to the relative luciferase activity of WT. Each experiment represents at least three sets of independent triplicates.

### Western blotting and immunodetection

Proteins were extracted from frozen cell pellets in RIPA buffer as described [9]. In order to inhibit de-SUMOylation of proteins, N-Ethylmaleimide (NEM) was added at 20 mM to lysis, Western blot (WB) and immunoprecipitation (IP) buffers. For direct immunoblotting, samples mixed with 4x Laemmli buffer were boiled for 5 min. Otherwise 10 μg of protein from each sample or immunocomplexes were submitted to 6% SDS-PAGE for IP, 7.5% SDS-PAGE for input and 12.5% SDS-PAGE for Ubc9 Western blot prior to immunoblotting. For immunoprecipitation experiments, 1 or 1.5 mg of total protein were preabsorbed with 20 μL protein G Sepharose 4 Fast Flow (GE Healthcare). Anti-3XFLAG M2 (1/500), anti-acetyllysine (1/1000) or anti-SUMO-1 (1/100) antibodies were mixed with 40 μL Protein G Sepharose beads for 1 h prior to an overnight incubation with the preabsorbed lysate supernatant at 4°C. The beads were then washed five times with lysis buffer (4 washings with RIPA, 1 washing with RIPA/NaCl 5M: 9/1, v/v) and finally 1 washing in NET-2 (50 mM Tris/HCl, pH 7.5, 150 mM NaCl, 0.05% Triton X-100) buffer. Proteins bound to the beads were eluted with 4X Laemmli buffer and analyzed by immunoblotting as above. Blots were first blocked in 5% non-fat milk for 1 h at room temperature prior being probed with primary antibodies (anti-3XFLAG M2, 1/2000; HA.11, 1/1000; anti-Ubc9, 1/1000; anti-His, 1/1000; anti-SUMO-1, 1/1000; anti-SUMO-2/3, 1/500; anti-acetyllysine, 1/1000; anti-GAPDH, 1/3000) overnight at 4°C and then probed with secondary anti-IgG antibodies conjugated to horseradish peroxidase (1/10000) for 1 h at room temperature before detection by chemiluminescence (ECL Advance or ECL Select, GE Healthcare Life Sciences). Each result in which immunoblots are presented corresponds to one representative experiment among at least three.

Antibodies against the 3xFLAG epitope (mouse monoclonal anti-FLAG M2 antibody, Sigma), HA epitope (mouse monoclonal HA.11 antibody 16B12, Covance Research Products), 6XHis epitope (mouse monoclonal anti-6XHis P5A11 antibody for WB, Biolegend; mouse monoclonal anti-His AD1.10 antibody for IP, Santa Cruz Biotechnology), SUMO-1 (rabbit monoclonal anti-SUMO-1, Millipore), SUMO-2/3 (rabbit polyclonal anti-SUMO-2/3, Millipore), Ubc9 (rabbit monoclonal antibody anti-Ubc9, Cell Signaling), acetyllysine (rabbit polyclonal anti-acetyllysine, ABCAM), GAPDH (rabbit polyclonal anti-glyceraldehyde-3-phosphate dehydrogenase antibody, Santa Cruz Biotechnologies) were used for immunoprecipitation and/or immunoblotting. Secondary antibodies conjugated to horseradish peroxidase were purchased from GE Healthcare Life Sciences. All the antibodies were used according to the manufacturer’s instructions.

### Densitometric and statistical analyses

The densitometric analysis was performed using the Quantity One v4.1 software (Bio-Rad, Hercules, CA) or *ImageJ* and statistical analyses were performed with PRISM 5 software (Graphpad, USA). M2 densitometric values were normalized to GAPDH and expressed as D^M2^/D^GAPDH^. Means were statistically analysed using the t-test or ANOVA and differences assessed at p<0.05 (*) or p<0.01 (**). SUMO-1 and acetyllysine densitometric values were expressed as D^Ac^/D^SUMO^ with the WT ratio in the siCtrl condition arbitrarily set as 100%.

## Results

### ΔLf possesses putative SUMO and acetylation sites and a putative SIMr motif

**Fig 1:**
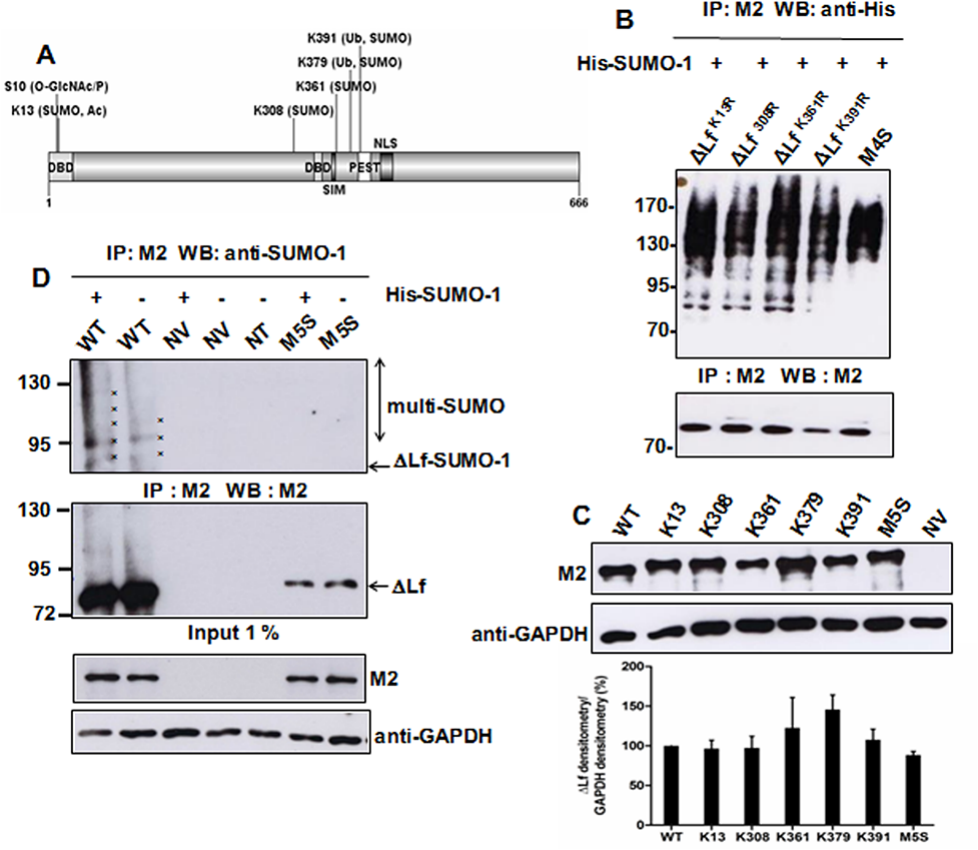
ΔLf is modified by SUMOylation. A) Schematic overview of ΔLf showing the NLS and PEST sequences, the two putative DBD and the putative SIM domain. The amino acid residues targeted by post-translational modifications are shown, S10 as the main *O*-GlcNAc/P site, K379 and K391 as the two ubiquitinated lysines, K13 as a putative acetylation site. B) Mutation of K13, K308, K361 and K391 individual lysine residues did not abolish ΔLf SUMOylation. The first series of ΔLf mutant constructs (ΔLf^K13R^, ΔLf^K308R^, ΔLf^K361R^, ΔLf^K391R^ and the M4S mutant constructs) were co-transfected with the pSG5-His-SUMO-1 (His-SUMO-1) plasmid in HEK-293 cells for 24 h prior to lysis. Lysates were immunoprecipitated with M2 and immunoblotted with anti-His antibodies and M2. The data presented correspond to one representative experiment of two conducted (n = 2). C) Expression of pCMV-3xFLAG-ΔLf^WT^ (WT) and the second series of SUMOylation mutant constructs. WT and the above constructs were transfected for 24 h prior to lysis. Whole cell extract was immunoblotted with either anti-FLAG M2 or anti-GAPDH antibodies. The data presented correspond to one representative experiment of at least seven conducted (n ≥ 7). NV: null vector (pCMV-3xFLAG). The level of expression of each mutant compared to WT is shown in the bar graph beneath the figure (n ≥ 7). D) ΔLf is SUMOylated and M5S is not. WT and the M5S mutant construct were co-transfected with or without the pSG5-His-SUMO-1 (His-SUMO-1) plasmid in HEK-293 cells for 24 h prior to lysis. Lysates were immunoprecipitated with M2 and immunoblotted with anti-SUMO-1 antibodies and M2. Asterisks correspond to SUMO bands (mono-SUMO, 86 kDa; multi-SUMO, 97, 108, 119 kDa). Lysates from HEK-293 cells transfected with a null vector (NV) and from non-transfected (NT) cells were used as negative controls. The data presented correspond to one representative experiment of at least three conducted (n ≥ 3).

*In silico* analysis of the ΔLf sequence with SUMOsp (http://sumosp.biocuckoo.org/) and SUMOplot (htpp://www.abgent.com/tools/sumoplot/) softwares revealed four lysine residues to be putative SUMO acceptors: K13 and K361 that are in the canonical ΨKXE/D motifs and K308 and K391 in non-canonical SUMO sequences (Table 1). The K13 consensus motif is of the PDSM-like type and the K308 motif of the NDSM-like type. K13 is within the first putative DBD and next to S10, the main *O*-GlcNAcylation/Phosphorylation site we previously demonstrated to control ΔLf transcriptional activity and stability (Fig 1A) [17]. Interestingly, K13 is also a putative acetylation site predicted using PAIL (prediction of acetylation on internal lysine [43] (http://bdmpail.biocuckoo.org/prediction.php). The other putative sites are concentrated in the central part of the protein, before the second DBD for K308, between this DBD and the PEST sequence for K361, and within the PEST sequence for K391 (Fig 1A).

**Table 1:** SUMO predictive motifs in human ΔLf.

SUMO motif^a^	Type^b^	Putative SUMO sites^c^
PDSM-like motif	ΨKXEXXSP	IK^13^RDSP
NDSM-like motif	ΨKXEXXEEEE	LRK^308^SEEE
Canonical consensus motif	ΨKXE	LK^361^GEA
Non consensus		YK^391^SQQSS

^a^SUMOsp (http://sumosp.biocuckoo.org/) and SUMOplot (htpp://www.abgent.com/tools/sumoplot/) softwares were used.

^b^The single-letter amino acid code is used.

^c^The numbering of the amino acid residues corresponds to human ΔLf.

In the case of a SUMO consensus motif, the target lysine is directly recognized by the conjugating enzyme Ubc9 whereas in the case of a non-consensus sequence, the recruitment of SUMO-loaded Ubc9 is realized *via* interactions with a SIM motif, which increases the modification of proximal lysine residues [30,44,45]. Thus, the prediction of SIM motifs using the GPS-SBM 1.0 User Interface software [46] reveals the presence of one SIM motif within the central region of ΔLf, near to the K361 site (Fig 1A). This motif is in a reversed orientation (SIMr) (Table 2) with four hydrophobic positions preceded by an acidic cluster and by a serine residue. Phosphorylation of SIM-associated serine residues is known to favor efficient recognition of SUMO [37,47]. Comparison of this motif with homologs from a number of vertebrate species reveals that it is conserved in Lf/ΔLf (Table 2). The functional significance of this motif and its role in ΔLf SUMOylation remain to be investigated.

**Table 2:** SUMO Interacting Motifs in human ΔLf and Lf from different species compared to the SIMr consensus (*D/E*)_3_ V/C L/V I/V V—*E* [37].

ΔLf/Lf	SUMO Interacting Motif^a^	Accession number
Human ΔLf^b^	^350^ S T T *E D* C **I** A **L V L** K G *E* ^363^	Q5EKS1
Bovine Lf^c^	^375^ S T T *D D* C **I V L V L** K G *E* ^388^	P24627
Goat Lf	^375^ S T T *D D* C **I** A **L V L** K G *E* ^388^	Q29477
Mouse Lf	^373^P T T *E D* C **I V** A **I M** K G *D* ^386^	P08071
Pig Lf	^371^ S T T *E D* C **I V** Q **V L** K G *E* ^384^	P14632
Horse Lf	^375^ S T T *E E* C **I** A **L V L** K G *E* ^388^	O77811
Sheep Lf	^375^ S T T *D E* C **I** A **L V L** K G *E* ^388^	AY792499
Camel Lf	^375^ S T T *E D* C **I** A **L V L** K G *E* ^388^	AJ131674

^a^The single-letter amino acid code is used; bold letters indicate the hydrophobic positions of the putative SUMO interacting motif, the acidic cluster is in italics, the SIM-associated serine residues are underlined.

^b^The numbering of the amino acid residues corresponds to human ΔLf.

^c^The numbering of the amino acid residues corresponds to Lfs.

The low abundance of ΔLf and the feeble percentage of SUMOylated conjugates rendered the detection of SUMOylated ΔLf and the subsequent mapping of its SUMO sites extremely difficult. Therefore we used a 3xFLAG-tagged ΔLf in order to detect it and we constructed a first series of SUMOylation mutants in which only one lysine residue was replaced at a time (ΔLf^K13R^, ΔLf^K308R^, ΔLf^K361R^, ΔLf^K391R^; S1 Table and S1 Fig left panel). Incubation of ΔLf and mutants with His-SUMO peptides caused the appearance of multiple higher molecular weight species indicative of SUMOylation events (Fig 1B). Moreover, mutation of each individual lysine residue did not abolish SUMOylation of the entire molecule (Fig 1B). Since competition between SUMO and ubiquitin ligases often occurs at ubiquitin sites, K379 which is the main ubiquitinated target on ΔLf [17] was also investigated. Thus, we produced a second series of mutant constructs in which only one putative SUMO site was preserved (S1 Table and S1 Fig right panel). We obtained five SUMO mutants named K13, K308, K361, K379 (which in fact corresponds to the M4S mutant) and K391, respectively and the M5S mutant in which all putative SUMOylation sites were abolished. ΔLf and its SUMOylation mutants were then expressed in HEK-293 cells which do not produce ΔLf endogeneously. We detected 3xFLAG-tagged ΔLf isoforms as a single band of the expected 75 kDa predicted molecular weight. The level of their expression was compared and Fig 1C shows that they were expressed at least at the same level as WT. K361 and notably K379 were expressed at a higher level than the other mutants but statistical analyses showed that these differences were not significant.

SUMOylation was first investigated on WT and M5S which were co-transfected with or without the SUMO-1 expression vector. An immunoprecipitation was then performed using the anti-FLAG antibody in order to specifically immunoprecipitate ΔLf or its SUMO variants. SUMOylation was then investigated using anti-SUMO-1 antibodies. Fig 1D shows that ΔLf was effectively SUMOylated and that SUMOylation was slightly increased when it was incubated with components of the SUMO pathway such as SUMO-1 (lane 1). Multiple higher molecular weight bands which may correspond to multi- or poly-ΔLf-SUMOylated forms were observed. Taking into account the *in silico* studies, this SUMO pattern (lanes 1–2) suggested that at least four SUMOylation sites are occupied (corresponding to 86, 97, 108 and 119 kDa, as shown by asterisks) for WT.

The feeble amount of SUMO-conjugates (Fig 1D, upper panel) compared to unmodified ΔLf (Fig 1D, middle panel) is in accordance with the literature. Thus, for most SUMOylated proteins, the levels of the SUMO forms are low relative to the unmodified form due to an efficient SUMOylation/deSUMOylation balance in cells [35].

M5S appeared not to be SUMOylated even when SUMO-1 was overexpressed suggesting that no other SUMO sites are present on the protein (Fig 1D, upper panel). Moreover, overexposure of this film failed to show additional bands that could suggest SUMOylation of the M5S construct (data not shown). Surprisingly, the M5S M2 immunoprecipitation signal is poor compared to the M5S M2 western blot signal. This could be due to the mutation of five lysine residues which may impair ΔLf conformation, resulting in poor immunoprecipitation even if IP was directed against 3XFLAG and not ΔLf itself.

### Mapping the main SUMO sites

**Fig 2:**
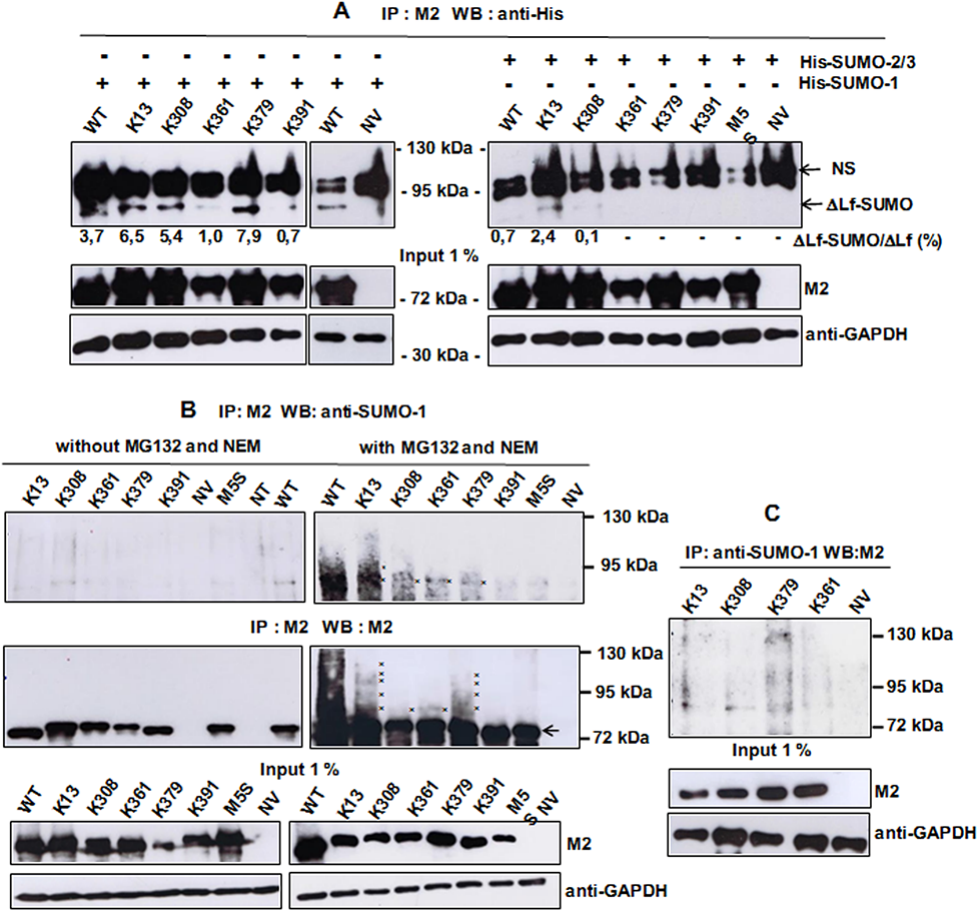
Mapping of the SUMO modification sites of ΔLf. A) K13, K308 and K379 are SUMO-1 acceptor sites. Cells were co-transfected by WT or the mutant constructs and pSG5-His-SUMO-1 or pcDNA3.1-His-SUMO-2/3 plasmids and then lysed 24 h later. Lysates were immunoprecipitated with M2 and immunoblotted with anti-His. Input was immunoblotted with either M2 or anti-GAPDH antibodies and used as loading control. The data presented correspond to one representative experiment of at least three conducted (n ≥ 3). B-C) WT and its mutants are SUMOylated *in vivo*. WT and the SUMO mutant constructs were transfected for 24 h prior to lysis. Whole cell extracts were immunoprecipitated with M2 and immunoblotted with either anti-SUMO-1 antibodies or M2 (B). Reverse immunoprecipitation was also performed (C). 1% of the cell extract (input) was immunoblotted with either M2 or anti-GAPDH antibodies used as loading control. Inhibition of proteasomal degradation was performed by incubating cells with MG132 for 2 h prior to lysis and inhibition of de-SUMOylation was performed by adding NEM to lysis, IP and WB buffers as in Material and Methods (Fig 2A and 2B right panels, and C). Data presented in Fig 2B (left panel) were obtained in the absence of proteasome and SENP inhibitors. Asterisks correspond to SUMO bands (mono-SUMO, 86 kDa; multi-SUMO, 97, 108, 119 kDa), the arrow corresponds to ΔLf. All the data presented correspond to one representative experiment of at least three conducted (n ≥ 3).

In order to identify SUMO acceptor sites, SUMO mutants were co-transfected with the His-SUMO-1 expression vector. A band corresponding to ΔLf-SUMO-1 forms is visible in each lane confirming that the five predicted sites were effectively SUMOylated (Fig 2A, left panel). K13, K308 and K379 are the three main acceptor sites. Surprisingly, the K361 mutant which possesses a predictive SUMO sequence that perfectly fits the optimal ΨKXE consensus sequence was poorly modified, as was K391. Overexpression or not of SUMO-1 did not modify the SUMO profile of the latter site confirming that it is not preferentially targeted by the SUMO machinery (Fig 2A and 2B). K391 is also ubiquitinated but it was not the main ubiquitin target site [17]. Interestingly, K379 which is the main ubiquitination site is also a good acceptor of SUMO, even though this lysine residue does not belong to a SUMO consensus sequence.

In order to investigate whether some of the SUMO-1 target sites might also be SUMO-2/3 acceptor sites, SUMO mutants were co-transfected with the His-SUMO-2/3 expression vector. Fig 2A (right panel) shows that SUMO-2/3 peptides might also bind K13 but the amount of the K13-SUMO2/3 form is feeble. Further work has to be done in order to confirm that this modification occurs as well *in vivo*. Control of specificity of His antibodies has been added since these antibodies revealed bands above 100 kDa even when His-SUMO-1 was not overexpressed (Fig 2A, upper left panel, lanes 7–10).

We next tried to evaluate whether ΔLf could be SUMOylated *in vivo*. The presence of SUMO proteases (SENPs) within the cells and also in cell extracts poses a significant problem for the detection of SUMOylated proteins. Therefore, we used N-Ethylmaleimide (NEM), which blocks cysteine proteases, as a SENPs inhibitor. Since NEM is not cell permeable we used it during cell lysis and immunoprecipitation steps. ΔLf-expressing cell lysates were immunoprecipitated with M2 and SUMO forms immunodetected with anti-SUMO-1 antibodies (Fig 2B, right panel). Slow migrating bands of strong intensity were detected for WT (upper panel) which might be due to the fact that WT is multi-SUMOylated. SUMO forms were also observed for K13, K308, K361 and K379 mutants (Fig 2B, upper right panel). Mutants that are unable to be ubiquitinated such as K13, K308 and K361 seem better in promoting SUMOylation of ΔLf (lanes 2–4). The K391 mutant seems poorly or even not modified, like the M5S mutant, which strongly suggests that only four of the five sites are modified by SUMO peptides.

Immunodetection by M2 showed the presence of a ladder of bands for WT, K13 and K379, confirming possible multi- and/or poly-modification (Fig 2B, middle right panel). A slow migrating band around 90 kDa was visible for K308 and K361 confirming the existence of a mono-SUMO-conjugated form of ΔLf. On the other hand, the strong ladder pattern (Fig 2B, middle right panel) observed for WT and K379 may also be partially due to ubiquitination as already described [17], suggesting that a competition between SUMO and ubiquitin modifications may occur. Since K13 is unable to be ubiquitinated, ladder bars might correspond to polySUMOylation. Therefore, M2 immunoprecipitation followed by SUMO-2/3 immunodetection of WT and SUMO mutant cell extracts was performed, but we were unable to observe SUMO-2/3 modifications on WT or its SUMO mutants (data not shown). Since K13 is also modified by SUMO-2/3 *in vitro* (Fig 2A, right panel), we will further investigate whether modification by SUMO-2/3 is relevant *in vivo*, then what impact a mixed SUMO chain could have on ΔLf activity and/or stability.

In order to confirm that endogeneous SUMOylation occurs on ΔLf we performed the same experiment in the absence of SENP and proteasome inhibitors. Fig 2A (left upper panel) effectively showed that no SUMOylation pattern was visible in those conditions.

Fig 2C corresponds to the reverse immunoprecipitation of K13, K308, K361 and K379 cell lysates with anti-SUMO-1 antibodies followed by M2 immunodetection. A faint band corresponding to ΔLf-SUMO-1 is visible and a poly-SUMO pattern is observed for K379. Since mixed SUMO/ubiquitin chains could be formed we will further investigate whether ΔLf might be modified at K379 by such a complex.

Collectively these results indicated that ΔLf could be SUMOylated at multiple lysine residues and that the band shift of ΔLf was indeed due to the covalent attachment of SUMO-1 with K13 as hotspot of SUMOylation.

### The SUMOylation/ubiquitination interplay at K379 controls ΔLf stability

**Fig 3:**
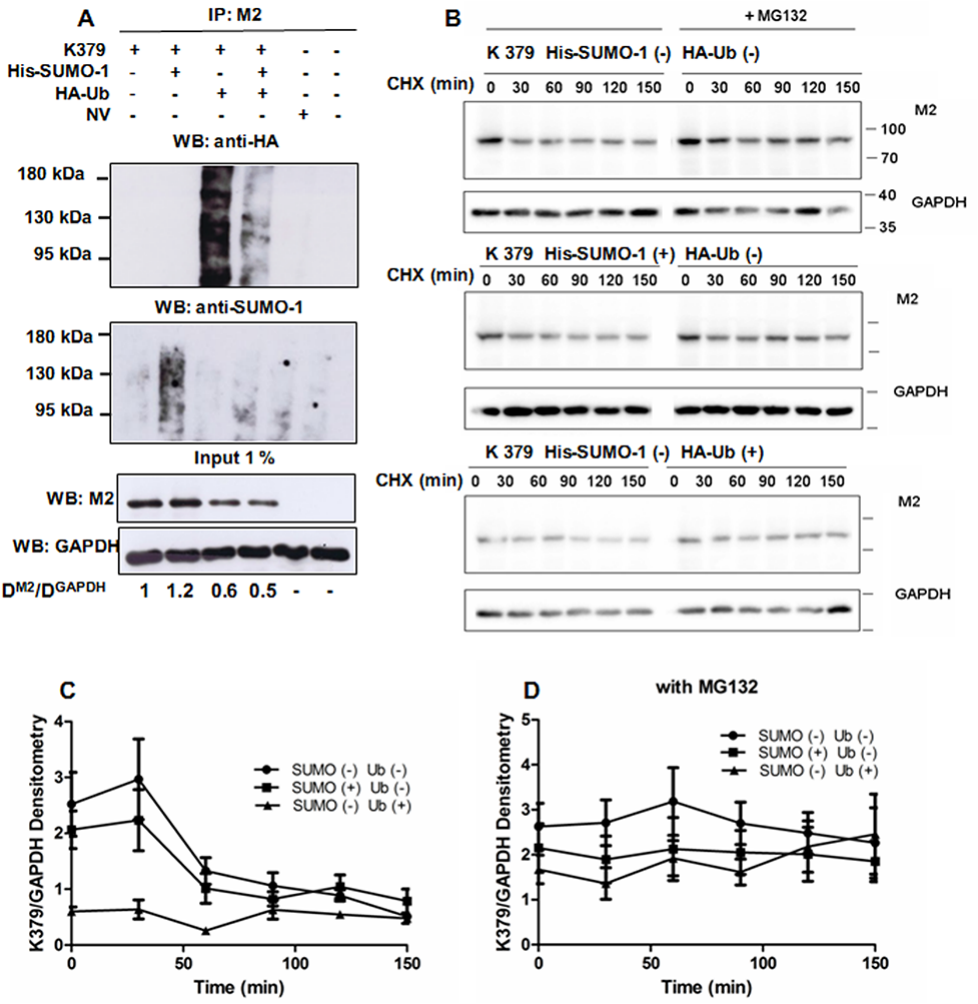
Competition between SUMOylation and ubiquitination at K379 controls ΔLf turnover. A) HEK-293 cells were co-transfected with K379 or NV constructs, His-SUMO-1 or/and HA-Ub-expression vectors for 24 h and then incubated with 10 μM of the proteasomal inhibitor MG132 for 2 h prior to lysis. NEM was added to lysis, IP and WB buffers. Total cell extracts were immunoprecipitated with M2 or used as input. Samples were immunoblotted with anti-HA (upper panel) or with anti-SUMO-1 (lower panel) antibodies. Input was immunoblotted with either M2 or anti-GAPDH antibodies and used as loading control. NS: non-specific. The data presented correspond to one representative experiment of at least three conducted (n ≥ 3). Lane 6 corresponds to non-transfected cells. B) Cells were transfected with K379, either with the His-SUMO-1 or the HA-Ub expression vector and then incubated with fresh medium supplemented by 10 μg.mL^-1^ CHX for the indicated time 24 h after transfection. K379 transfected cells were incubated without (left panel) or with (right panel) 10 μM MG132 for 2 h prior to lysis. Total protein extracts were immunoblotted with either M2 or anti-GAPDH antibodies. Detection was carried out using a Fusion SOLO camera (Vilbert Lourmat). The data presented (B) correspond to one representative experiment of at least five conducted. C-D) The M2 densitometric analyses are normalized for the matching GAPDH immunoblots and expressed as ratio D^K379^/D^GAPDH^ as described in Materials and Methods. Data are shown as the means ± SD (n = 5).

Since, as for many transcription factors, ΔLf is rapidly degraded, we previously demonstrated that its turnover was dependent on both the Ub-proteasome pathway and the *O*-GlcNAc/phosphate interplay [17]. We also characterized K379 as the major site for ΔLf poly-ubiquitination. Since K379 is also targeted by SUMO ligases we next investigated whether a crosstalk exists between the ubiquitin and SUMO pathways. Fig 3A shows that a ladder of polyubiquitinated K379 forms is visible in the presence of recombinant HA-Ubiquitin (upper panel, lane 3). The intensity of the polyubiquitination signal decreases when SUMO-1 peptides are overexpressed (Fig 3A upper panel, lane 4). After stripping, the immunoblot was revealed using anti-SUMO-1 antibodies (middle panel). Increased SUMOylation could be observed for the K379 mutant when SUMO-1 was overexpressed (Fig 3A, middle panel, lane 2) as already shown for WT in Fig 1D. We also observed a decrease in this SUMO signal in the presence of recombinant ubiquitin (Fig 3A, middle panel, lanes 2 and 4). Loadings of K379 (input) confirmed that in the presence of recombinant ubiquitin the expression level of K379 is lower than in the untreated condition or when SUMO-1 is overexpressed. These data support the view that K379 is indeed the target of an ubiquitin/SUMO switch.

We next investigated whether this interplay acts on K379 stability. To measure the K379 turnover rate indirectly, we performed incubations (0–150 min) with cycloheximide (CHX), a potent inhibitor of *de novo* protein synthesis. The K379 (left panels 1, 3 and 5) and GAPDH (left panels 2, 4 and 6) contents of HEK-293 cells were analysed following addition of CHX (Fig 3B). GAPDH was used as an internal control. Differences in the steady state levels of the K379 mutant were readily apparent after 30 min, which may correspond to the delay necessary for observing the first effects of treatment. HA-Ubiquitin (HA-Ub) or His-SUMO-1 (SUMO-1) expression vectors were co-transfected or not in HEK-293 cells with the K379 construct (Fig 3B). Densitometric data are expressed as D^K379^/D^GAPDH^ ratio as described in Materials and Methods. HA-Ub overexpression led to an overall 3-fold decrease in K379 stability compared to His-SUMO-1 treated cells (Fig 3C) confirming that increasing ubiquitination drives ΔLf to degradation as we showed previously [17]. When K379-expressing cells overexpressed the SUMO-1 peptide, the stability of the mutant was comparable to that of untreated K379 mutant. Nevertheless, the fact that we did not observe a strong protection as may be expected against proteosomal degradation may be due to the fact that the modified pool of K379 is very feeble (less than 10%) even when SUMO-1 is overexpressed (Fig 2A). The same experiment was conducted with MG132 (right panels) and densitometric results showed that K379 is very stable when the degradation of ubiquitin-conjugated proteins is reduced, whatever the applied treatments (Fig 3D). Taken together these results confirmed, as already described for other substrates, that SUMOylation may antagonize ubiquitination at K379 and hence positively affect the proteolytic stability of ΔLf.

### SUMOylation of ΔLf represses its transcriptional activity

**Fig 4:**
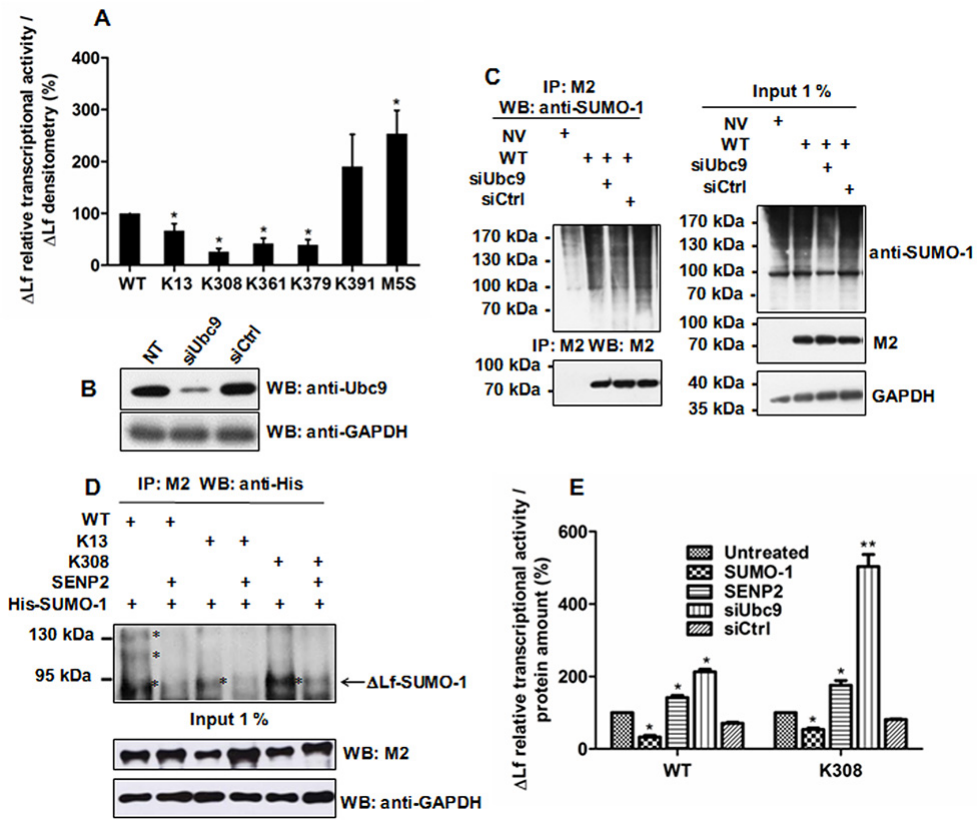
SUMOylation of ΔLf represses its transcriptional activity. A) Cells were co-transfected with pGL3-S1^Skp1^-Luc reporter vector and WT, SUMO mutant constructs or null vector in order to assay the relative transcriptional activity of WT and its SUMO mutants. Relative luciferase activities are expressed as described in Materials and Methods (n≥5; p < 0.05 (*)). B-E) Alteration of SUMOylation at K308 modulates ΔLf transcriptional activity. B) Knockdown of Ubc9 was performed using siUbc9/siCtrl as described in Materials and Methods and followed after 48 h of incubation by immunoblotting of the cell extracts with either anti-Ubc9 or anti-GAPDH antibodies. C) Knockdown of Ubc9 leads to a decrease in SUMOylation. Lysates were immunoprecipitated with M2 and immunoblotted with anti-SUMO-1 or M2. Input was immunoblotted with M2, anti-SUMO-1 or anti-GAPDH antibodies and used as controls. D) Deconjugation of SUMO-1 from WT, K13 and K308 by SENP2. HEK-293 cells were co-transfected with WT or the K308 construct together with pSG5-His-SUMO-1 and pcDNA-SENP2-SV5 and then lysed 24 h later. Lysates were immunoprecipitated with M2 and immunoblotted with anti-His. Input was immunoblotted with either M2 or anti-GAPDH antibodies. C-D) Cells were incubated with MG132 for 2 h prior to lysis and NEM added to lysis, IP and WB buffers. The data presented correspond to one representative experiment of at least six conducted (n ≥ 6) (B) and to one representative experiment of at least two conducted (n ≥ 2) (C, D). E) Cells were co-transfected with pGL3-S1^Skp1^-Luc reporter vector, either WT or the K308 construct together with pSG5-His-SUMO-1 or pcDNA-SENP2-SV5. Relative luciferase activities were also assayed in Ubc9 invalidated cells. HEK-293 cells were reverse transfected for 24 h using siRNAs targeting Ubc9 (siUbc9) or a scrambled control sequence (siCtrl) before being transfected as described above to evaluate the relative transcriptional activities of ΔLf and the K308 mutant. Relative luciferase activities are expressed as described in Materials and Methods (n≥5; p < 0.05 (*), p < 0.01 (**)).

**Fig 5:**
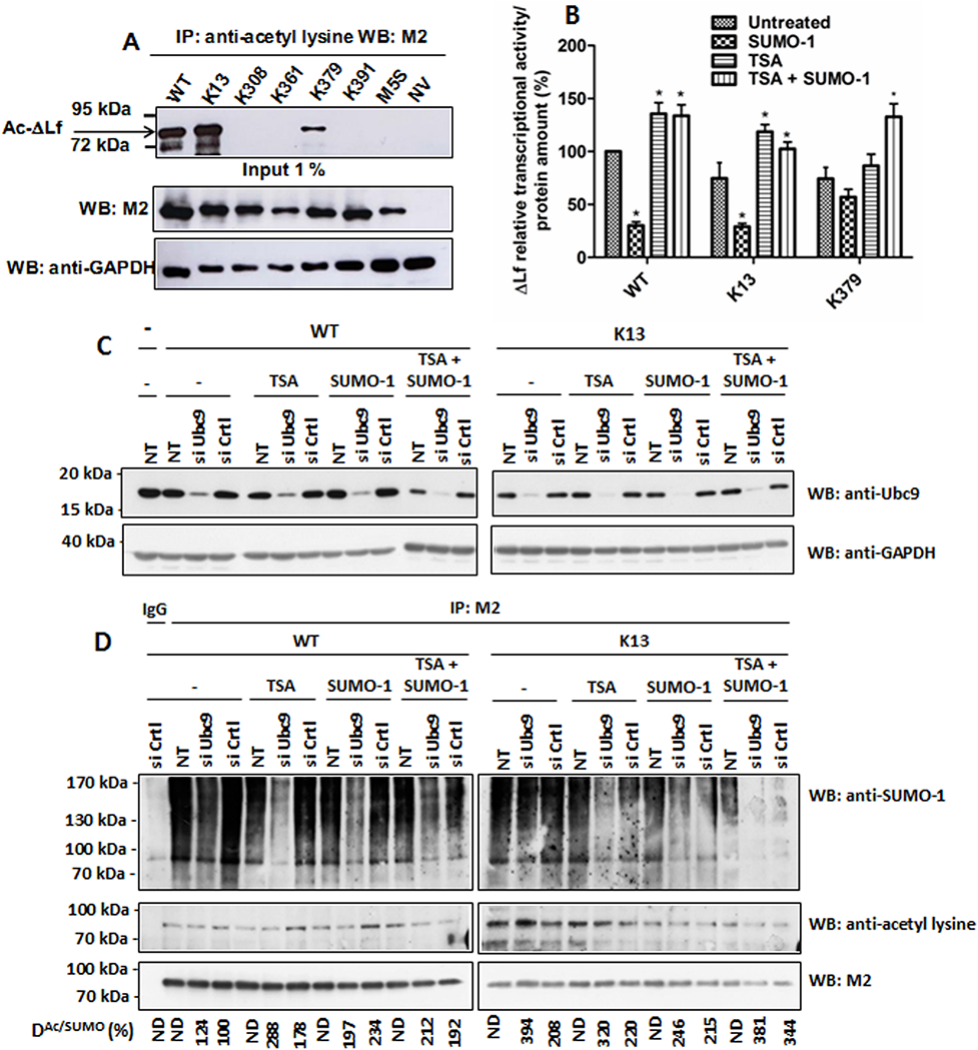
A SUMOylation/acetylation switch at K13 controls ΔLf transcriptional activity. (A) K13 is the main acetylation site. Cells were co-transfected by WT, the mutant constructs or the null vector and then lysed 24 h later. Lysates were immunoprecipitated with anti-acetyllysine antibodies and immunoblotted with M2. Input was immunoblotted with either M2 or anti-GAPDH antibodies and used as loading control (n = 3). B) Relative transcriptional activity of K13 and K379 mutants compared to WT. Cells were co-transfected with pGL3-S1^Skp1^-Luc reporter vector and WT, K13 or K379. His-SUMO-1 expression vector and/or the deacetylase inhibitor Trichostatin A (TSA, 15 ng/mL) were used to modulate the acetylation/SUMOylation ratio. Relative luciferase activities are expressed as described in Materials and Methods (n≥3; p < 0.05 (*)). C-D) Modulation of the SUMOylation level was performed either by knocking down Ubc9 using siUbc9 or by overexpressing His-SUMO-1 peptides (SUMO-1). Cells were reverse transfected or not with RNAiMax using 5 nM of siUbc9/siCtrl for 24 h before being transfected for 24 h with WT or K13 plasmid with or without His-SUMO-1 plasmid. Before cell lysis, the acetylation level was altered or not by an overnight treatment with TSA (15 ng/mL). Cells were then incubated with 10 μM of the proteasomal inhibitor MG132 for 2 h prior to lysis. NEM was added to lysis, IP and WB buffers. (C) Input was immunoblotted with anti-Ubc9 (upper panel) or anti-GAPDH (lower panel) antibodies. (D) Samples were immunoprecipitated with M2 and immunoblotted with anti-SUMO-1 (upper panel), then with anti-acetyllysine (middle panel) or finally with M2 (lower panel) antibodies. The acetylation/SUMOylation ratio (R^Ac/SUMO^) was assayed as described in Material and Methods. The data presented correspond to one representative experiment of two conducted.

To study the physiological consequences of ΔLf SUMOylation, we next assayed the transcriptional activity of the mutants compared to wild type (Fig 4A). SUMOylation usually triggers recruitment of corepressors such as HDACs, which condense chromatin and prevent transcription. We used a luciferase reporter construct driven by a basal promoter and one ΔLf response element present in a fragment of the *Skp1* promoter [9]. In this experiment, the cells were not co-transfected with His-SUMO-1, so the status of ΔLf SUMOylation completely relied on endogenous SUMO activity. The 2.5-fold increased transcriptional activity of the SUMOylation-null mutant confirmed that SUMOylation negatively regulates ΔLf transcriptional activity. Since M5S could not be SUMOylated, over-expression of this mutant without co-expression of SUMO-1 justified the conclusion drawn here that the SUMOylation of ΔLf is part of a regulatory event that governs its activity. The small amount of ΔLf-SUMO forms present in cells could not account for the 2.5 fold increment observed in the transcriptional activity induced by the M5S mutant compared to WT. This has been already described for numerous transcription factors and suggests that SUMOylation is required to initiate transcriptional repression but not to maintain it [19,48]. We then compared the activity of mutants in which only one SUMOylation site was preserved to that of M5S in order to evaluate the impact of adding only one regulatory site at a time. The K391 mutant, which is poorly modified, showed transcriptional activity nearly comparable to that of M5S (Fig 4A) suggesting that the presence of SUMO on this site does not crucially regulate ΔLf transcriptional activity. In contrast, the transcriptional activities of the K308, K361 and K379 mutants were strongly inhibited, by 10-fold for K308 and by nearly 6 fold for the other sites compared to M5S, and by 4 fold for K308 and by around 2–2.5 fold for the other two sites compared to WT, suggesting that these three sites are important for regulation. K361, which is poorly SUMOylated, is nevertheless strongly involved in the repression process. The transcriptional activity of the K13 mutant also decreases but to a lesser extent, by 3.5-fold compared to M5S and by 1.5-fold compared to WT. This may be due to the SUMO/acetylation switch discussed below (Fig 5). The transcriptional activity of the SUMO mutants K13, K361, K379 and notably K308, is lower than that of WT. This may be due to the fact that ΔLf is multi-SUMOylated and that the distribution of SUMO conjugates at each site leads to a “dilution” of the effect in the WT compared to the mutants with only one SUMO acceptor site, which may be more heavily SUMOylated.

Since SUMO modifications on the K308 site led to the highest inhibitory impact on ΔLf transcriptional activity, we next focused our attention on K308 and investigated the impact of altering its SUMO pattern. Therefore we increased SUMOylation by using the His-SUMO-1 expression vector and decreased it by performing either de-SUMOylation using recombinant SENP2 protease or knockdown using specific short interfering RNA against Ubc9 (siUbc9). Prior to performing transcriptional activity assays, we first showed that siUbc9 efficiently invalidated Ubc9 expression (Fig 4B) leading to a decrease in the level of SUMOylation of both ΔLf (Fig 4C, IP, lane 3) and other protein substrates (Fig 4C, input, lane 3). Immunoprecipitation of ΔLf or K13- and K308-expressing cell lysates with M2 followed by immunodetection of SUMO forms using anti-His antibodies (Fig 4D) showed that effective de-SUMOylation was produced in the presence of recombinant SENP2 and is visible in the second, fourth and sixth lanes compared to the untreated condition. Fig 4E shows that overexpression of the His-SUMO-1 peptide strongly decreased the transcriptional activity of ΔLf and its K308 mutant whereas overexpression of SENP2 protease significantly increased it. Moreover, siRNA-mediated depletion of endogenous Ubc9 abolished the repressive potential of SUMOylation and led to a drastic activation of the reporter gene activity (Fig 4E). This combination of overexpression and knockdown experiments demonstrate that SUMOylation plays an important role in ΔLf-mediated transcriptional activation. Collectively, these results showed that effectively SUMOylation negatively controls ΔLf transcriptional activity and may convert ΔLf into a transcriptional repressor.

### Acetylation attenuates SUMO-mediated transcriptional repression

Acetylation regulates numerous cellular processes, including the regulation of transcription [49,50]. Acetylation of internal lysine residues is a reversible PTM which strongly alters the electrostatic properties of its targets, modulating their functions, such as protein-protein interactions, DNA binding, activity, stability and subcellular localization [22]. Since competition between SUMOylation and acetylation occurs on many substrates and a putative acetylation site was found on ΔLf at K13, we next investigated whether the SUMO sites might also be acceptors for acetyltransferases. We performed mapping of ΔLf acetylation sites by western blotting of the different expressed mutant ΔLfs using an anti-acetyllysine antibody. Among the five SUMOylated lysine residues, K13 and K379 were the only acetylation acceptor sites with K13 being the major acetylated residue (Fig 5A, lanes 2 and 5, respectively). Mutation of these two lysine residues in the other SUMO mutants (Fig 5A, lanes 3, 4 and 6) and in M5S (Fig 5A, lane 7) resulted in a complete loss of the acetylation signal suggesting that only two acetylation sites are present on ΔLf. These data also confirmed the existence of a possible interplay between acetylation and SUMOylation for K13 and suggested an acetylation/SUMOylation/ubiquitination crosstalk for K379.

We next assayed the impact of the SUMO/acetylation interplay on ΔLf-mediated transactivation (Fig 5B). We increased either SUMOylation by overexpressing SUMO-1 peptide or raised the acetylation level by using the HDAC inhibitor Trichostatin A (TSA). TSA-induced acetylation was able to promote ΔLf- and K13-mediated activation by nearly 1.5 fold compared to the untreated condition and 4-fold compared to the condition when SUMO forms were overexpressed (SUMO-1). These data suggested that dynamic interactions between these two posttranslational modifications may occur. The fact that the enhanced WT and K13 transcriptional activities due to TSA-induced acetylation were not modified when SUMO-1 peptides were overexpressed (TSA+SUMO-1) may be due to the fact that acetylation is a less labile PTM than SUMOylation. Indeed, SENPs have to be inhibited in order to observe SUMO forms whereas we do not need to inhibit HDACs in order to visualize acetylated forms. That modulation of the SUMO or acetylation pattern on the K379 mutant had less impact on ΔLf transcriptional activity may be due to the fact that ubiquitination also targets this site.

In order to confirm these results we further investigated whether an increase in the levels of acetylation may result in a reduction in the levels of ΔLf SUMOylation and conversely (Fig 5C and 5D). Therefore, we invalidated Ubc9 or inhibited deacetylases with TSA in order to increase acetylation levels, or raised the levels of SUMOylation by overexpressing His-SUMO-1 peptides (SUMO-1), and assessed the impact on ΔLf acetylation. Results shown in Fig 5C confirmed that siUbc9 were functional. More than 80% of the Ubc9 protein disappeared 48 h post-transfection as compared to untreated (NT) or siCtrl treated cells. Moreover, knockdown was not sensitive to treatment with TSA or to the overexpression of His-SUMO-1. Immunoprecipitation of ΔLf-expressing cell lysates with M2 was followed by immunodetection of SUMO-1, acetylated forms or ΔLf (Fig 5D). SUMO profiles of WT and its K13 mutant, when Ubc9 was invalidated, confirmed a decrease in SUMOylation compared to controls (Fig 5D, lane 3 left panel and 2 right panel, respectively) which was more pronounced after TSA treatment (lanes 6 and 12 left panel and lanes 5 and 11 right panel). Overexpression of SUMO-1 peptides together with siUbc9 treatment did not lead to increased SUMOylation of WT and K13 as expected (Fig 5D, lane 9 left panel and 8 right panel, respectively) compared to their respective controls (Fig 5D, lanes 8 and 10 left panel and 7 and 9 right panel, respectively). Thus, ΔLf SUMOylation levels were downregulated following Ubc9 knockdown and when inhibition of HDACs was achieved with TSA. We also analyzed the modification of the acetylation profile of WT and K13 in the above conditions but we did not observe visible variations. Therefore we assayed the acetylation/SUMOylation ratio. This ratio varied slightly when WT was expressed in siUbc9 cells but increased by nearly 2-fold when these cells were grown overnight in the presence of TSA and 3-fold in siUbc9-TSA-treated HEK-293 cells. These data are in accordance with the literature and confirmed increased acetylation when HDACs are inhibited by TSA. Overexpression of SUMO-1 peptides in siCtrl versus siUbc9 cells leads to a comparable acetylation/SUMO ratio. When cells overexpressing SUMO-1 were treated with TSA, acetylation was favoured. The same experiment was conducted with the K13 mutant. The acetylation/SUMOylation ratio was 2-fold higher than WT and rose 4-fold in Ubc9-null cells suggesting that the K13 mutant with only one acetylation/SUMOylation site may preferentially exist as an acetylated form. TSA treatment led to an increased acetylation/SUMOylation ratio as expected. Taken together these results suggest that acetylation antagonizes SUMOylation and may downregulate SUMO effects at K13 (Fig 5B and 5D). The crosstalk between these sites could constitute part of the «ΔLf code » responsible for the control of the transactivation of ΔLf target genes.

## Discussion

Transient PTMs like acetylation, phosphorylation, *O*-GlcNAcylation, ubiquitination and SUMOylation are fast and efficient ways for the cell to respond to different stimuli. Transcription factors are often regulated by combinations of these different PTMs which might act as a molecular barcode [51]. In this report, we demonstrated that ΔLf, known to be modified by *O*-GlcNAcylation, phosphorylation at S10 and ubiquitination at K379 and K391 [17], can also be modified by SUMOylation and acetylation. We provide experimental evidence that SUMOylation represses ΔLf transcriptional activity whereas acetylation increases it. Moreover, by competing with ubiquitination, SUMOylation influences positively ΔLf stability.

Considering the fact that, for a given protein, only a small fraction is commonly found in the SUMOylated state and that this modification is transient, it was difficult to visualize or isolate endogeneous SUMO forms. Nevertheless we were able to observe SUMO-1 isoforms of ΔLf *in situ*. The ΔLf SUMOylation pattern, manifested as multiple bands, is consistent with the presence of multiple SUMOylation sites on the protein. We identified five SUMOylation sites among which the K13 and K361 sites conform to the consensus sequence of ΨKXE/D. Furthermore, SUMOylation was also mapped at K308, K379 and K391, which are non-canonical sites. Among these, K13, K308 and K379 are the three SUMO hotspots.

SUMOylation of transcription factors generally antagonizes their activation potential or mediates repression, although in a few cases SUMOylation has been associated with reciprocal effects resulting in activation. Whereas SUMOylation decreases the transactivation potential of c- Jun and the androgen receptor [52,53], it increases heat shock factor-2 (HSF2) transactivation capacity [54]. SUMOylation can both positively and negatively modulate p53 transcriptional activity depending on the target promoter [55]. Here we demonstrated that SUMOylation of ΔLf represses its transcriptional activity using a fragment of the *Skp1* promoter containing one ΔLfRE. ΔLf binds to and transactivates *DcpS*, *Skp1*, *Bax*, *SelH*, *GTF2F2* and *UBE2E1* genes [9,12–14] through similar consensus response elements. Nevertheless it may be interesting to investigate whether ΔLf differentially transactivates its target genes depending on its SUMOylation status.

Since ΔLf possesses five SUMO target sites it is difficult to determine whether each of the individual sites has a specific role. Moreover, three of them are targeted by other PTMs, rendering this study even more complex. In order to establish whether any single SUMOylation site was important for the transactivation capacity we compared the activities of mutants disabled specifically at each individual consensus SUMOylation site. The transactivation capacity of each single-site lysine mutant was similar or slightly increased compared to WT (data not shown). Since multiple sites contribute to the control of ΔLf transactivation capacity, the loss of only one SUMO site has only small effects on the activity of ΔLf suggesting either that individual sites act in a redundant manner or that SUMOylation at multiple sites is necessary. Therefore we next studied the transcriptional activity of each mutant in which only one SUMO site was preserved. The K308 mutant strongly inhibited ΔLf transcriptional activity. Moreover when SUMOylation was impaired either after Ubc9 knockdown or in the presence of increased expression of the SENP2 protease, the transcriptional activity of K308 was increased 5-fold compared to that of the K308 mutant expressed in untreated cells. This activity strongly decreased by nearly 2-fold when SUMO-1 peptides were overexpressed. These results demonstrated that SUMOylation at K308 strongly controls ΔLf activity, which may be due to the fact that the region downstream from its SUMO motif is rich in acidic residues as in NDSM. NDSM interacts twice with Ubc9, first between the consensus motif and the active site of the enzyme and also between the acidic tail of the consensus and the basic patch of Ubc9 [27]. Thus, the NDSM acidic patch plays an important role in determining the efficiency of substrate SUMOylation which consequently results in enhanced transcriptional repressive properties.

Our *in silico* studies led us to discover a reverse putative consensus SIMr motif in the vicinity of the K361 SUMO site which is conserved among mammalian species (Table 1). SIMs, which mediate non-covalent interactions between SUMO and SIM-containing proteins [56], can mediate SUMO modification of numerous proteins, resulting in changes in their activity. Moreover a serine residue that is proximal to this SIMr might be the target of kinases as described for non-histone proteins such as PML, EXO9 and PIAS proteins [47]. The presence of a SIMr and/or a phospho-SIMr might be essential to enhance interactions with a SUMO protein and mediate SUMO conjugation. Therefore, the functionality of such a motif has to be established for ΔLf.

SUMOylation usually competes with ubiquitination, phosphorylation and acetylation. Ubiquitination/SUMOylation and SUMOylation/acetylation are mutually exclusive whereas SUMOylation/phosphorylation can be agonistic or antagonistic depending on the substrates. The dialogue between SUMO and the other modifications is emerging as a common mechanism that allows control of the transcriptional activity of transcription factors [21]. Two of the SUMO sites are targeted by acetyltransferases. Acetylation is also a dynamic process which mainly contributes to activation of transcription factors [57,58]. Thus, K13 and K379 are acetylated with K13 as the major acetylation site. Modulation of the SUMO/acetylation status has a strong impact on K13 transcriptional activity. In this way, SUMO/acetylation modification of ΔLf could act as a form of switch for the selective interaction with corepressor or coactivator partners, thus modulating ΔLf activity from a transcriptional repressor/corepressor to a coactivator. This is consistent with literature data. Thus, it was shown that SUMOylation inhibits MEF2, HIC1 and KLF8 transcriptional activities whereas acetylation blocks these inhibitory effects [39,40,59,60]. This acetylation/SUMOylation switch is regulated by phosphorylation for MEF2 [39] and it will be interesting to investigate whether ΔLf acetylation/SUMOylation interplay is also controlled by phosphorylation events. The K13 site has a SUMOylation motif close to PDSM motifs [28]. Phosphorylation of the SP motif within this consensus sequence plays an important role in promoting SUMOylation of several substrates including MEF2A [28,39]. Therefore we will have to investigate whether S16 might be of potential functional importance in the regulation of SUMOylation at K13. Moreover, since at the N-terminus, the K13 SUMO/acetylation site is adjacent to the S10 *O*-GlcNAcylation/phosphorylation site we will further investigate whether the *O*-GlcNAc/Phosphate interplay interferes with the SUMOylation/acetylation switch or acts in parallel. The crosstalk between these sites may constitute the ΔLf code responsible for the control of the transactivation of ΔLf target genes. We know from our results [17] and from the literature that acetylation and phosphorylation both lead to transcriptional activation whereas *O-*GlcNAcylation and SUMOylation repress it. So we hypothesize that this region might be part of the ΔLf transactivation domain which has never been identified. On the other hand, Ubc9 itself is acetylated and its acetylation leads to its decreased binding to NDSM substrates, causing a reduction in their SUMOylation status. Therefore Ubc9 acetylation/deacetylation may serve as a dynamic switch for NDSM substrates such as the K308 site in order to control their SUMOylation [61].

K379 and K391 could be both SUMOylated and ubiquitinated. SUMOylation competes with ubiquitination and positively regulates ΔLf stability. Indeed, SUMO frequently influences protein stability by blocking ubiquitin attachment sites [19,36]. K379, which is the main target of both the SUMO and ubiquitin machineries, does not possess a SUMO consensus sequence but is located in the vicinity of the PEST region. It has been shown that Ubc9 could directly interact with the PEST region of SUMO-1 target proteins such as HIPK2 [62] but this is not always the case. It will be interesting to determine the Ubc9-interacting region of ΔLf and investigate whether it overlaps the PEST region. The ubiquitination/SUMOylation interplay exerts a critical role in the maintenance of cellular homeostasis by controlling the turnover of numerous of proteins and notably transcription factors. The switch between these two PTMs needs to be tightly regulated in a spatiotemporal manner and other PTMs, such as phosphorylation, contribute to regulate the ubiquitin/SUMO pathways. PEST sequences are rich in S/TP motifs and are often recognized and phosphorylated by proline-directed S/T protein kinases [63]. Phosphorylation can prevent or favor SUMO-1 conjugation as previously shown for IκBα [36], c-Jun [52] and p53 [52]. We already showed that the ΔLf PEST motif contains three serine residues (S392, S395 and S396) which are phosphorylated prior to ubiquitination of the targets K379 and K391 in their vicinity. Mutation of these two lysine residues or of the three serine residues (S392, S395 and S396) within the PEST motif strongly increased the half-life of ΔLf [17]. Moreover, we showed that they were equivalent phosphorylation targets due to their proximity. Therefore, at the PEST motif, phosphorylation and ubiquitination work in synergy [17], while SUMOylation and ubiquitination are antagonistic PTMs. Therefore this crosstalk could constitute the ΔLf code responsible for the control of ΔLf stability.

This regulation is driven by the *O*-GlcNAc/phosphate interplay at S10. *O*-GlcNAc coordinately regulates ΔLf stability and transcriptional activity. The pool of ΔLf may exist under a stable but not functional *O*-GlcNAc isoform. Since the level of *O*-GlcNAc changes during the cell cycle or is altered, such as in tumorigenesis, deglycosylated ΔLf will become the target of kinases leading to its activation and polyubiquination [1,17]. ΔLf is at the crossroads between cell survival and cell death. It triggers cell cycle arrest and apoptosis *via* the transactivation of several crucial target genes. Therefore, modifications of their expression may have marked consequences and, depending on cell homeostasis, their transactivation by ΔLf should be transiently suspended. In this context, the SUMOylation/acetylation switch at K13 acts as a second level of control. The activation of the SUMO pathway leads to repression of ΔLf transcriptional activity whereas acetylation, by counteracting SUMOylation at gene promoters, restores it. Increasing evidence shows that *O*-GlcNAcylation not only interferes with phosphorylation but also crosstalks with other PTMs including acetylation [64], methylation [64,65], ubiquitination [66,67] and poly-ubiquitination [68,69]. However crosstalk with SUMOylation has not yet been reported and we are currently investigated the *O*-GlcNAcylation/SUMOylation interrelationship.

In conclusion, we showed that SUMO modification provides subtle, context-dependent, regulatory input to modulate ΔLf target gene expression. Moreover, we confirmed that ΔLf, like many transcription factors, is regulated by combinations of different PTMs which act as a molecular barcode. Thus, cooperation and/or competition between SUMOylation, ubiquitination, acetylation, phosphorylation and *O*-GlcNAcylation may contribute to the establishment of a fine regulation of ΔLf transcriptional activity depending on the type of target gene and cellular homeostasis. In this paper, we have focused on the role of SUMOylation but it has not escaped our attention that lysine residues can also be methylated and that such modifications can also affect the activity and stability of proteins such as p53 [70]. Further studies of the roles of PTMs in the molecular mechanisms of ΔLf functions are warranted.

## Supporting Information

S1 Table Name of mutant constructs, location of amino acid modifications and oligonucleotides used for mutagenesis. (DOCX)

S1 Fig Schematic representation of the two series of ΔLf SUMO mutant constructs. (TIF)
