# Supplementary material for: Modification by SUMOylation Controls Both the Transcriptional Activity and the Stability of Delta-Lactoferrin
Source: PLoS One. 2015 Jun 19;10(6):e0129965. doi: 10.1371/journal.pone.0129965 (PMC4474976; doi:10.1371/journal.pone.0129965)
Supplement: S1 Table — (DOCX) [file pone.0129965.s001.docx]

**Table S1**: Name of mutant constructs, location of amino acid modifications and oligonucleotides used for mutagenesis.

| **Name of mutants** | **Mutated amino acid(s)** | **Site-directed mutagenesis oligonucleotides** |
| --- | --- | --- |
| ΔLf^K13R^ | K13R | F: 5’-CTCCTGTCAGCTGCATACGGAGAGACTCCCCCATC-3’  R: 5’-GATGGGGGAGTCTCTCCGTATGCAGCTGACAGGAG-3’ |
| ΔLf^K308R^ | K308R | F: 5’-CATCCAGAACTTGAGGAGAAGTGAGGAGGAAGTGG-3’  R: 5’-CCACTTCCTCCTCACTTCTCCTCAAGTTCTGGATG-3’ |
| ΔLf^K361R^ | K361R | F: 5’-ACTGCATCGCCCTGGTGCTGAGAGGAGAAGCTGAT-3’  R: 5’-ATCAGCTTCTCCTCTCAGCACCAGGGCGATGCAGT-3’ |
| ΔLf^K379R^ | K379R | F: 5’-TGTGTACACTGCATGCAGATGTGGTTTGGTGCCTG -3’  R: 5’-CAGGCACCAAACCACATCTGCATGCAGTGTACACA-3’ |
| ΔLf^K391R^ | K391R | F: 5'-CCTGGCAGAGAACTACAGATCCCAACAAAGCAGTG-3’  R: 5’-CACTGCTTTGTTGGGATCTGTAGTTCTCTGCCAGG-3’ |
| K13 | K308R, K361R, K379R, K391R |  |
| K308 | K13R, K361R, K379R, K391R |  |
| K361 | K13R, K308R, K379R, K391R |  |
| K379 | K13R, K308R, K361R, K391R |  |
| K391 | K13R, K308R, K361R, K379R |  |
| M5S | K13R, K308R, K361R, K379R, K391R |  |
