# Supplementary figures and images for: Modification by SUMOylation Controls Both the Transcriptional Activity and the Stability of Delta-Lactoferrin
Source: PLoS One. 2015 Jun 19;10(6):e0129965. doi: 10.1371/journal.pone.0129965 (PMC4474976; doi:10.1371/journal.pone.0129965)

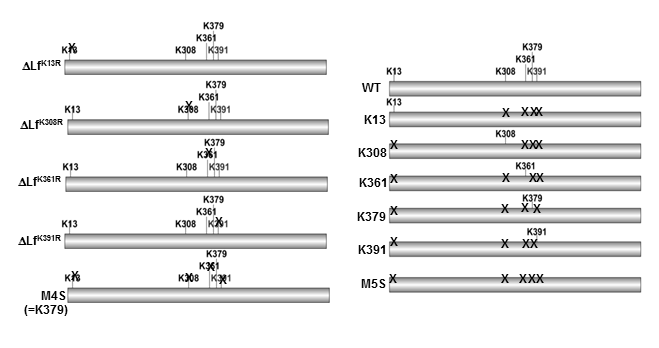

Supplement: S1 Fig — (TIF) [file pone.0129965.s002.tif]
